# Synthesis of ZIF-67/CoX-LDH-Derived Composites Through Cation Engineering Strategy: The Electromagnetic Wave Absorbers with Dielectric–Magnetic Loss Synergy

**DOI:** 10.3390/molecules30224386

**Published:** 2025-11-13

**Authors:** Aixiong Ge, Anqi Ju, Shaobo Qu

**Affiliations:** 1 Department of Basic Sciences, Air Force Engineering University, Xi’an 710051, China; 2 State Key Laboratory for Advanc Fiber Materials, College of Materials Science and Engineering, Donghua University, Shanghai 201620, China; anqiju@dhu.edu.cn

**Keywords:** electromagnetic wave interference, ZIF-67, interface polarization, cation engineering

## Abstract

Electromagnetic wave interference has escalated into a pervasive global issue, driving intensified research efforts across both civilian and military domains. However, the development of advanced electromagnetic wave (EMW) absorbers with finely tunable dielectric and magnetic loss properties has emerged as a pivotal strategy for mitigating electromagnetic pollution. Herein, we propose a cation engineering strategy to tailor the absorption properties of ZIF-67-derived layered double hydroxide (LDH) composites through systematic substitution of Co^2+^ with Fe, Mn, Zn, or Ni and stoichiometric control (Co/X = 1:4, 1:1). Mn/Zn doping enhances dipole polarization via lattice distortion, while structural analysis confirms that higher Co/X ratios preserve core–shell architectures, optimizing impedance matching. In contrast, Fe incorporation leads to excessive conductivity and impedance mismatch. The optimized CoNi1-4 composite exhibits superior broadband absorption (EAB = 4.52 GHz at 1.8 mm thickness, RL_min_ = −24.5 dB), attributed to synergistic interface polarization and magnetic coupling. This study delivers a highly tailorable materials platform that enables a deeper fundamental understanding of the synergy between dielectric and magnetic loss processes, thereby offering new pathways for optimizing electromagnetic wave absorption.

## 1. Introduction

Electromagnetic wave (EMW) absorbing materials have become indispensable in addressing electromagnetic pollution across civilian and military sectors, particularly in stealth technology and high-frequency communication systems [1,2,3,4]. The escalating demand for broadband absorption capability, lightweight architectures, and tunable dielectric-magnetic synergy drives continuous innovation in absorber design. Traditional ferrites and carbon-based composites, while effective, often suffer from impedance mismatch and narrow absorption bandwidth due to limited structural tunability. This has prompted exploration of layered double hydroxides (LDHs) as precursors for constructing hierarchical absorbers through controlled pyrolysis. LDH materials are widely investigated and used in a variety of studies, including catalysis [5,6,7], supercapacitors [8,9,10], water purification [11,12] and, from another point of view, ion recoveries [13,14]. Also, lots of usages were produced in biology and physiology, such as cancer therapy [15] and other applications [16,17,18,19,20,21]. Multiple methods like coprecipitation [22,23], ion exchange [24,25,26], and hydrothermal [27,28] can lead to the controllable formation of LDHs.

Recent advances highlight the potential of MOF/LDH-derived composites, where pyrolysis generates porous carbon matrices embedded with transition metal oxides. This porous structure always possesses a quite low density, which is very helpful in their applications. Owing to the efficient dielectric loss and magnetic loss brought by metal oxides along with the conductive loss attributes to the carbon species, these products can perform well in absorbing electromagnetic waves. These metal oxide/carbon framework mixtures were widely studied. Ren et al. demonstrated Zn-regulated interfacial polarization in CoZn-MOF derivatives through temperature-dependent phase evolution [29]. They explained the presence of Zn at a rather lower pyrolysis temperature would introduce more interfacial polarization loss and provide better properties. Wu et al. achieved enhanced conductive loss by integrating CoNi-LDH nanoflowers with carbon fibers, though without pyrolysis-induced structural transitions [30]. Particularly noteworthy are core–shell ZIF-67@LDH architectures, where Wu et al. optimized Fe doping ratios in ZIF-67 to achieve −67.3 dB reflection loss while maintaining structural integrity [31]. By calcinating the CoNi LDH tubular cyanuric acid melamine precursor, the CoNi carbon hybrid was investigated by Hu et al., and −35.6 dB was reached [32]. Other mixtures like NiFe, CoMo, and many other mixtures were also vastly investigated [33,34,35]. Systematic comparisons of different transition metal elements (Fe, Mn, Zn, and Ni) within such systems remain limited, creating significant knowledge gaps in several critical areas as follows: (i) the element-specific role in crystalline phase evolution during pyrolysis, (ii) the quantitative relationship between cation ratios and dielectric/magnetic loss mechanisms, and (iii) the synergistic interplay between carbon conductivity and oxide-derived magnetic properties.

To address these challenges, we implement a cation engineering strategy utilizing ZIF-67@CoX-LDH precursors (X = Fe, Mn, Zn, Ni) with controlled Co/X stoichiometries of 1:4 and 1:1. This approach enables a systematic investigation of how metal identity and concentration govern the following: (1) the preservation of morphology during thermal transformation, (2) the introduction of lattice defects and associated polarization, and (3) the optimization of impedance matching through balanced permittivity and permeability. Our comparative analysis reveals that Mn and Zn doping primarily enhance dipole polarization, whereas Ni doping fosters magnetic coupling. These findings provide critical insights for the rational design of next-generation electromagnetic wave absorbers with tailored performance.

## 2. Results and Discussion

**Figure 1:**
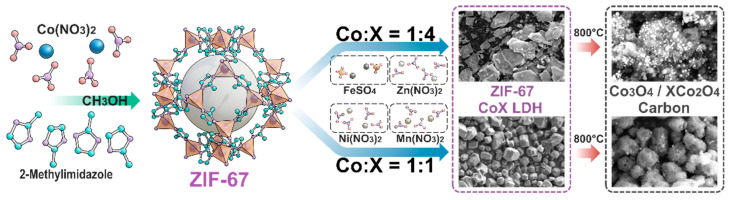
Synthesis of ZIF-67/CoX-LDH derived composites.

As shown in Figure 1, ZIF-67 nanoparticles simultaneously act as both the hard template for the ZIF-67/CoX-LDH composite material and the cobalt source for CoX-LDH, laying the foundation for the distribution of elements and substances in the final derivatives. In the synthesis process of composite electromagnetic wave-absorbing materials, ZIF-67 reacts with Fe/Mn/Zn/Ni salts at two different molar ratios (1:4 and 1:1) to form CoFe/CoMn/CoZn/CoNi LDHs precursors. Then, these powders were pyrolyzed at 800 °C for 30 min under an argon atmosphere in a tube furnace. After high-temperature calcination treatment, the core ZIF-67 would turn into porous carbon frameworks containing Co_3_O_4_, while the outer CoX LDHs (X = Fe/Mn/Zn/Ni) would turn into their oxides as the ferrite frameworks. The substances and their molar ratio were shown in Table 1.

**Figure 2:**
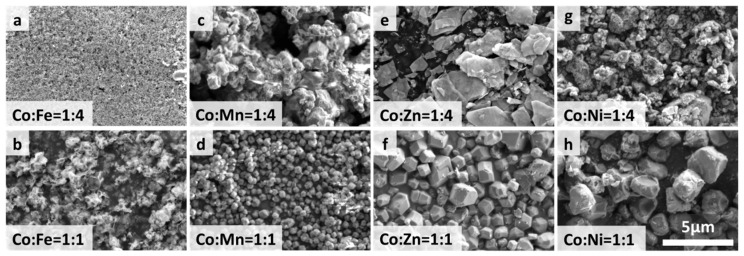
SEM images of LDH materials before pyrolysis.

**Figure 3:**
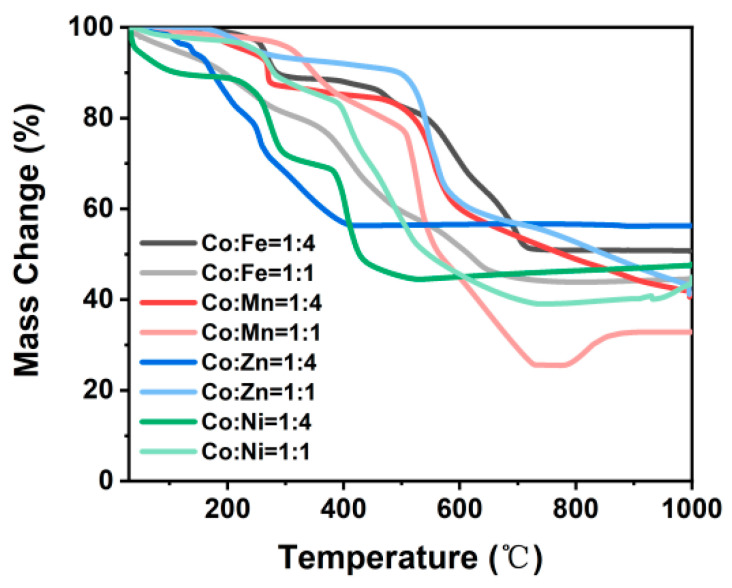
Thermogravimetry analysis of each sample during pyrolysis process.

**Figure 4:**
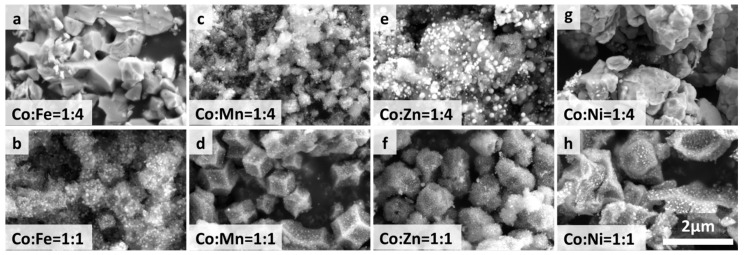
SEM images of ZIF-67/CoX-LDH derived composites.

**Figure 5:**
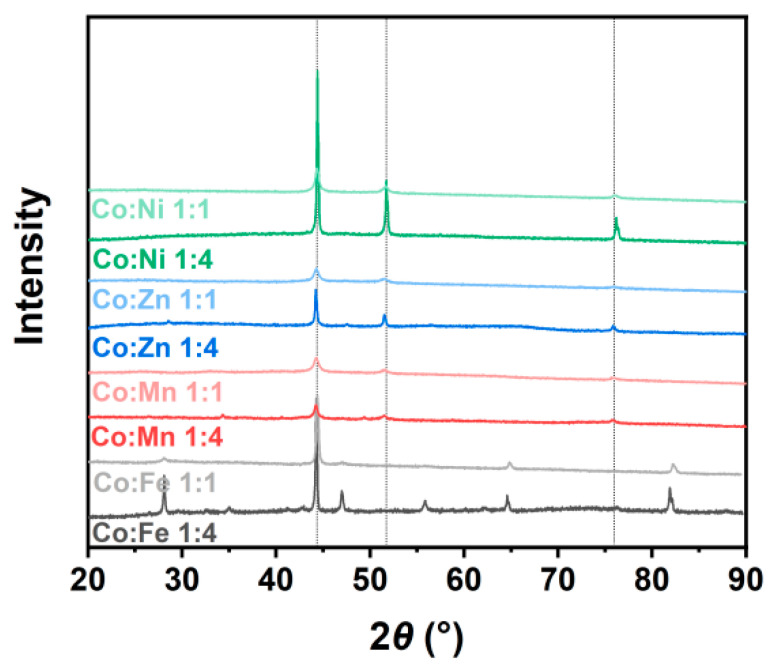
XRD curves of ZIF-67/CoX-LDH derived composites.

The synthesized ZIF-67/CoX-LDH derived composites exhibit different microstructures and substance distributions (Figure 2). According to the thermogravimetric analysis curve data as shown in Figure 3, after all samples were heated to 800 °C, the weight no longer changed further, indicating that the carbonization process of ZIF-67 and LDH was basically completed: the organic framework was transformed into a stable carbon skeleton, while the metal elements were converted into stable corresponding metal compounds. According to Figure 4, CoFe11, CoMn11, CoZn11, and CoNi11 have more regular shapes, while their CoX14 partners are more amorphous. As the Co/X ratio increases, that means less Co in ZIF-67 frameworks was replaced by other ions. Therefore, the frameworks themselves were less destroyed, so the microstructures were maintained after pyrolysis. Similar results were also reported by Wu, et al. [31]. XRD was also characterized from 20 to 90° and displayed in Figure 5. All powder XRD curves possess 2θ = 44.2°, 51.5° and 75.6° peaks, corresponding to (111), (200), and (220) faces of cobalt. And CoFe samples have some extra peaks at 44.8°, 64.9°, 82.3°, corresponding to (110), (200), and (221) faces of α-iron, respectively. Peak at 47.0° may correspond to the (101) face of ε-cobalt (h.c.p.), as it is more stable than α-cobalt (f.c.c.) at room temperature [36]. Peaks at 28.1° and 55.8° corresponding to (002) and (004) faces of graphite, which may form under the catalysis of Fe. Different from CoFe, the other six samples exhibit only three main peaks at almost the same position. For the CoMn sample, Mn could substitute Co from the lattice; a very small shift in the unit cell was reported [37,38]. As for zinc, part of it may vaporize under 800 °C calcination; others may form single atoms and enhance the EMW absorption property [39]. In the end, there may be a homeomorphism between Co and Ni. So, there are no extra peaks in CoNi samples, and the intensities of their three main peaks are much higher [8].

**Figure 6:**
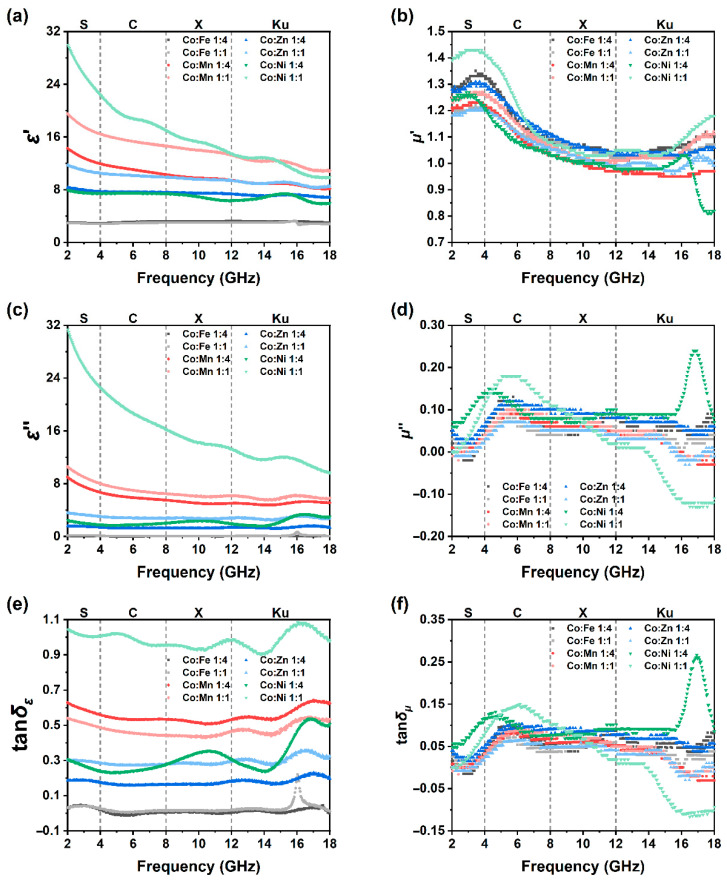
Permittivity and permeability of ZIF-67/CoX-LDH derived composites. (**a**) Real part of complex permittivity, (**b**) imaginary part of complex permittivity, (**c**) permittivity loss, (**d**) real part of complex permeability, (**e**) imaginary part of complex permeability, (**f**) permeability loss.

Complex 2. GHz. Also, dielectric and magnetic loss tangents were calculated (tan *δ_ε_* = *ε*’’/*ε*’, tan *δ_μ_* = *μ*’’/*μ*’), results are shown in Figure 6. As demonstrated in Figure 6a,c, for both the real part and the imaginary part of permittivity, CoX11 is also higher than CoX14. The real part of complex permittivity (*ε*’) of two CoFe samples stabilizes around 3, as the imaginary part (*ε*’’) is almost equal to 0. For CoMn14 and CoMn11, *ε*’ descents from 14 and 20 at 2 GHz to around 10 and 13 at 18 GHz, respectively. Their *ε*’’ mainly stabilizes at about 8 to 12 and slowly decreases with increasing frequency. Similar regularity can be found in CoZn and CoNi samples as well.

The introduction of other ions damaged the regular Co lattice, so the polarization of bulk materials becomes much more disordered, and this causes the *ε*’ decrease. Also, as the frequency increases, the polarization can hardly catch the electric field vibration, *ε*’ also decreases naturally. As the Co lattice is damaged by other ions, its conductivity also decreases, and this results in the decrease in ε’’. Notice that at about 16 GHz, *ε*’’ of CoFe11 has a small sharp peak, which is much more apparent at tan *δ_ε_* (Figure 6e). CoFe11 shall have a resonance at this specific frequency. Yet the permittivity and permeability of iron species (like cobalt ferrite, Fe_3_O_4_, and carbonyl iron) have been exanimed, even simulated thoroughly, and no peak was observed around 16 GHz [40,41,42,43]. This peak may come from a specific Fe species.

As shown in Figure 6e, for the CoFe sample, the tangent of the loss angle in the range of 2 to 18 GHz is concentrated around 0. For the CoMn sample, the tangent of the loss angle is mainly around 0.6. For the CoZn sample, the tangent of the loss angle is mainly around 0.2 and 0.3. However, for the CoNi sample, the tangent of the loss angle fluctuates significantly. For CoNi14 and CoNi11, it is mainly 0.3 and 1.0 in the S, C, and X bands, and the fluctuation is more remarkable in the Ku band.

As depicted in Figure 6b, the real parts of the permeability of the eight samples exhibit similar patterns. In the S band, it is approximately 1.2 to 1.4, then decreases to around 1.0 to 1.1 in the C band, and remains basically the same in the X and Ku bands. However, the imaginary parts of the permeability are different. As shown in Figure 6d, the permeabilities of the eight samples are all around 0 in the S band, and increase to 0.05 to 0.10 in the C band. The permeability of the CoFe continues to maintain this value in the Ku band. In contrast, the permeabilities of the CoMn and CoZn decline rapidly in the Ku band, dropping to −0.15 and −0.10, respectively, at 18 GHz. CoNi14 and CoNi11 show a peak and a gradual decline, respectively. Since the tangent of the magnetic loss angle is mainly affected by the imaginary part, tan *δ_μ_* follows a pattern similar to that of the imaginary part of the permeability (Figure 6f).

**Figure 7:**
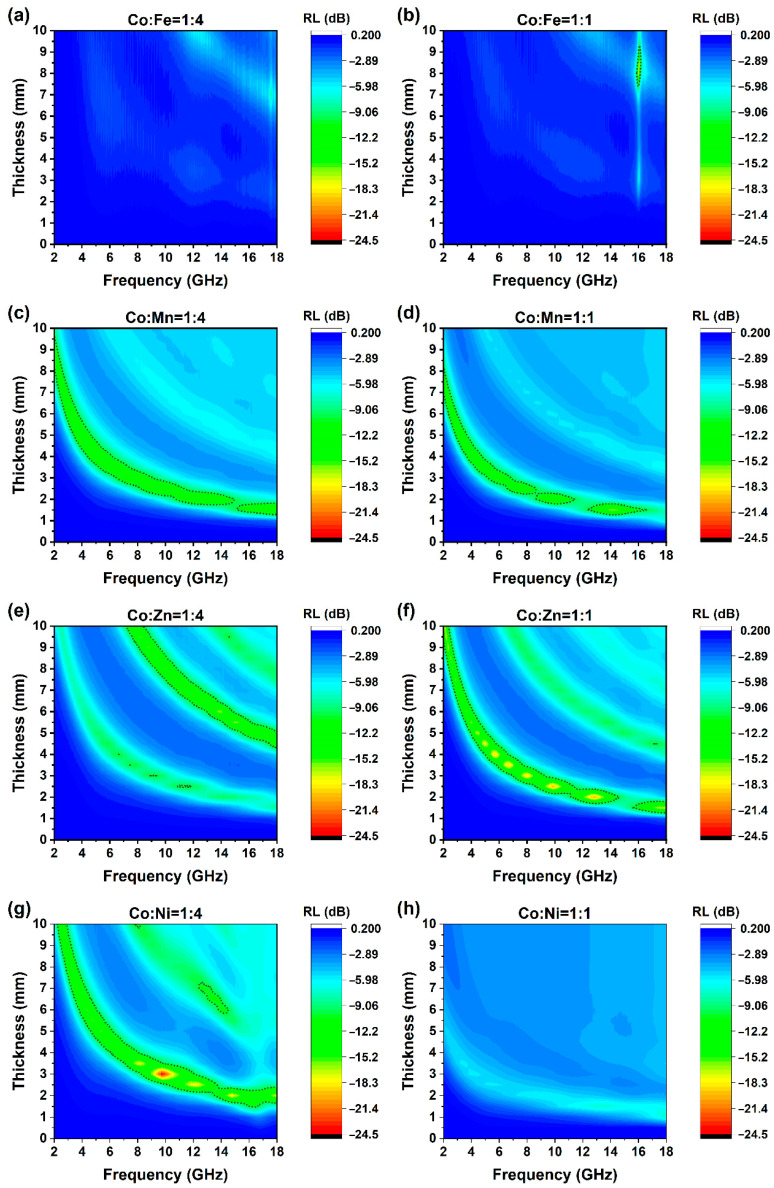
Reflection loss of ZIF-67/CoX-LDH derived composites at different thickness. Dashed line marks RL = −10 Db, (**a**) Co:Fe = 1:4; (**b**) Co:Fe = 1:1; (**c**) Co:Mn = 1:4; (**d**) Co:Mn = 1:1; (**e**) Co:Zn = 1:4; (**f**) Co:Zn = 1:1; (**g**) Co:Ni = 1:4; (**h**) Co:Ni = 1:1.

A coaxial probe was used to determine the EMW absorbance properties of all samples. Reflection loss of different frequencies at different thicknesses was also calculated. As shown in Figure 7a–h, when thicknesses were below 1 mm, no apparent RL could be observed. When the thickness increases to more than 1 mm, as the thickness increases, the reflection attenuation peaks gradually migrate from the high-frequency end (18 GHz) to the low-frequency band (2 GHz). The maximum effective absorption bandwidth (EAB) of all materials is concentrated in the Ku band, and the corresponding thickness is about 1.5–2.0 mm.

Specifically for these eight groups, under the ratios of 1:4 and 1:1 for CoFe, there is almost no obvious attenuation. The maximum attenuation of CoFe14 is only −8.85 dB. For CoFe11, only at a thickness of 8.0 mm and in the high-frequency band around 16 GHz, the peak sharply lower to less than −10 dB. On the other hand, the CoMn and CoZn samples demonstrate excellent wave-absorbing properties, presenting broad reflection attenuation valleys. Specifically, the overall performance of CoMn11 is marginally superior to that of CoMn14. Although the contour map of CoMn14 appears more continuous, the maximum reflection attenuation of CoMn11 attains −16.7 dB, and its EAB is slightly broader than that of CoMn14.

For the CoZn samples, three relatively prominent absorbing bands are observed, extending from the lower-left to the upper-right region. Notably, the positions of these bands remain essentially identical for the two ratios. However, the primary absorbing band of CoZn14 corresponds to the second band, while for CoZn11, it is the first band. Significantly, the EAB of CoZn11 is considerably larger than that of CoZn14. Moreover, the peak value of CoZn11 reaches −20.44 dB, which is substantially greater than the −16.76 dB of CoZn14. Finally, the CoNi samples exhibit a rather unique pattern. On one hand, the CoNi14 has an EAB of 4.52 GHz, and its attenuation peak value of −24.5 dB is conspicuously remarkable among all eight groups of samples. On the other hand, CoNi11 does not display evident wave absorbing characteristics, with its attenuation peak value being merely −7.60 dB. Details were summarized in Table 2.

**Figure 8:**
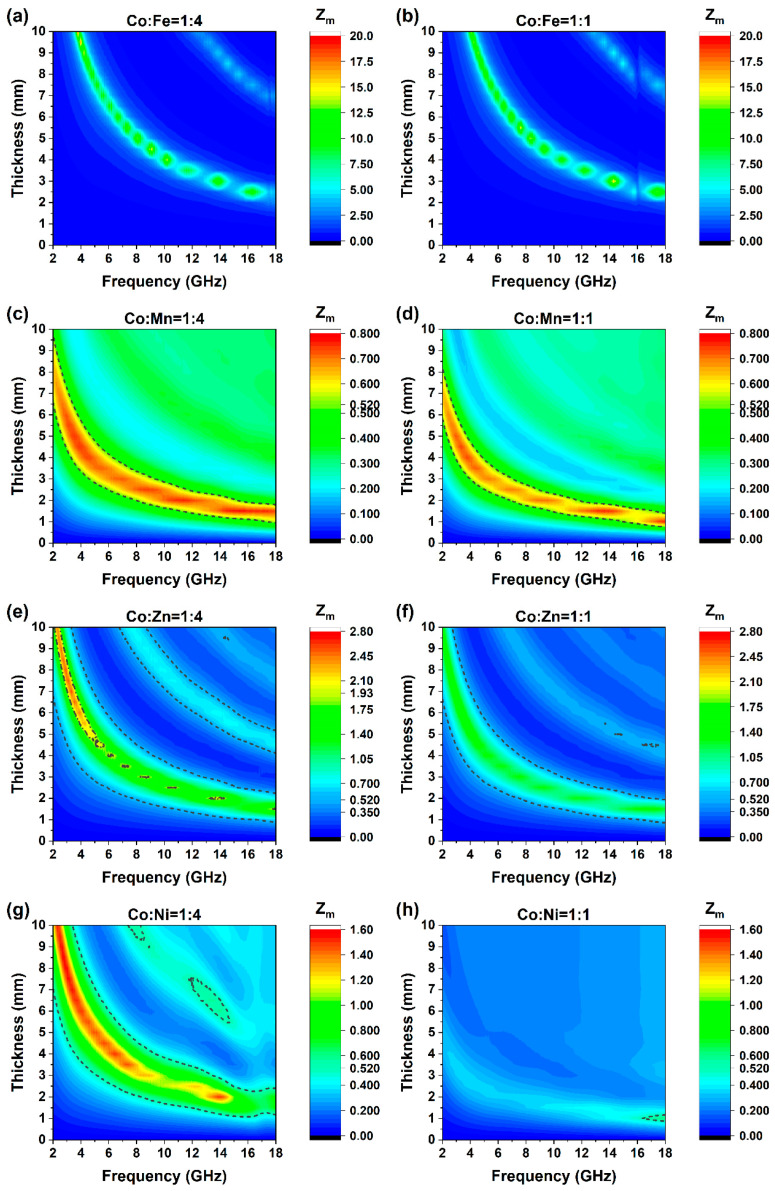
Impedance matching of eight LDH materials. Notice that the legends are not on the same scale for different combinations, (**a**) Co:Fe = 1:4; (**b**) Co:Fe = 1:1; (**c**) Co:Mn = 1:4; (**d**) Co:Mn = 1:1; (**e**) Co:Zn = 1:4; (**f**) Co:Zn = 1:1; (**g**) Co:Ni = 1:4; (**h**) Co:Ni = 1:1.

Impedance matching (IM) of the LDH materials was calculated simultaneously and displayed in Figure 8a–h. For an effective absorption criterion, that is −10 dB, a well matching shall be recognized when IM is between 0.52 and 1.93 [44,45]. According to this criterion, the optimal impedance-matching range of CoMn can cover the entire 2–18 GHz band, and it is consistent with the RL test results of CoMn. Overall, the range of CoMn14 exceeds that of CoMn11, and its maximum bandwidth is indeed larger than that of CoMn11. Meanwhile, CoNi14 also has the same impedance matching range and covers a sufficiently large area. However, the value of CoNi11 is almost entirely below the determination range of 0.52. This implies that electromagnetic waves (EMW) can hardly penetrate CoNi11, which also fully corresponds to its RL test results (Figure 7h).

**Figure 9:**
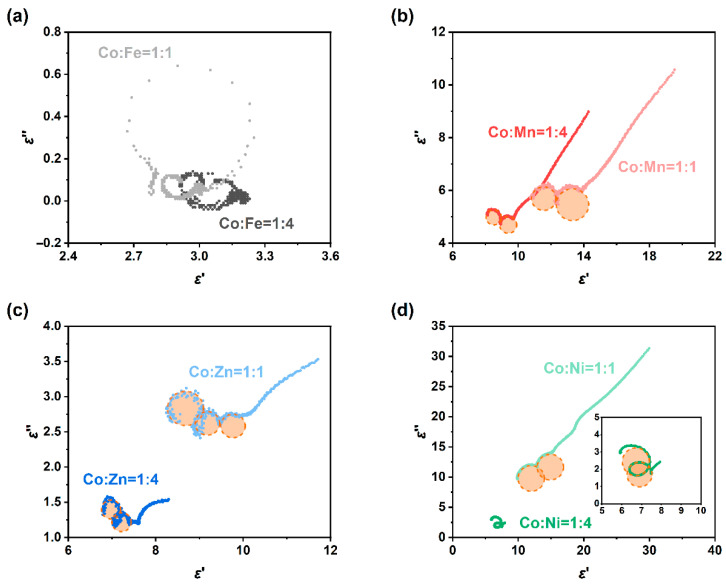
Cole-Cole curves of ZIF-67/CoX-LDH (X=Mn and Zn) derived composites, (**a**) Co:Fe = 1:1; (**b**) Co:Mn = 1:1; (**c**) Co:Zn = 1:1; (**d**) Co:Ni = 1:1.

**Figure 10:**
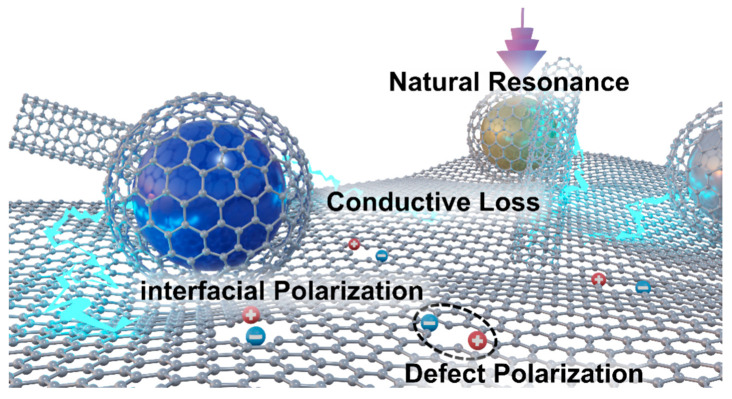
Possible schematic diagram of electromagnetic dissipation mechanism.

The Cole-Cole curves of the permittivity of the eight samples are shown in Figure 9. The CoFe sample only exhibits two very small loss circles (note the coordinate scale), and perhaps due to oxidation, there is no obvious conductivity tail (Figure 9a). In contrast, CoMn (Figure 9b) and CoZn (Figure 9c) show distinct loss circles and long tails. In particular, the CoMn sample has two significant polarization relaxation loss circles. The long tails of both correspond to the high conductivity loss of the carbon part. Both the real and imaginary parts of the permittivity of CoNi11 are very large, but the “central angle” of its loss circle is very small, mainly showing conductivity loss. However, the “central angle” of the loss circle of CoNi14 is much larger (insert of Figure 9d), indicating that its loss impedance matching is better, and thus its wave-absorbing performance is also remarkable. The results of the Cole–Cole curves are in good agreement with the wave absorbing conditions in Figure 7. A possible electromagnetic wave absorption mechanism of ZIF/CoX-LDH derived composites is illustrated in Figure 10, in which natural resonance, conductive loss, interfacial polarization, and defect polarization together contribute to the wave absorption effect of these materials.

## 3. Experimental

### 3.1. Synthesis of ZIF-67

A total of 4 mmol of Co(NO_3_)_2_·6H_2_O was dissolved in 60 mL of methanol under five-minute ultrasonication to facilitate complete dissolution. The resulting solution was then slowly introduced into 20 mL of a methanol solution containing 2-methylimidazole (1.2 mol/L) and stirred for 30 min to form ZIF-67. The mixture was subsequently centrifuged, and the solid product was washed three times with ethanol. The collected ZIF-67 precipitate was dried under vacuum at 70 °C for 12 h.

### 3.2. Synthesis of ZIF-67/CoX-LDH Materials

All four ZIF-67/CoX-LDH materials (where X = Fe, Mn, Zn, Ni) were synthesized following analogous procedures. ZIF-67 served as both the template and cobalt source, reacting with Fe, Mn, Zn, or Ni salts at two molar ratios (Co/X = 1:4 and 1:1) to form the corresponding CoFe-, CoMn-, CoZn-, and CoNi-LDH precursors.

For instance, in the synthesis of ZIF-67/CoNi-LDH, 200 mg of as-prepared ZIF-67 (≈0.688 mmol) was dispersed in 20 mL of an ethanol solution containing Ni(NO_3_)_2_·6H_2_O (0.688 mmol). The reaction proceeded under stirring at 300 rpm for 30 min. The product was then collected by centrifugation, washed three times with ethanol, and the resulting ZIF-67/CoX-LDH precipitate was dried under vacuum at 70 °C for 12 h.

### 3.3. Synthesis of ZIF-67/CoX-LDH Derived Composites

All precursor samples were subjected to pyrolysis in a tube furnace (Hefei Kejing Material Technology Co., Ltd., Hefei, China) under an argon atmosphere. The temperature was raised to 800 °C at a heating rate of 5 °C/min, held for 30 min, and then allowed to cool naturally to room temperature. The resulting powder composites were collected for subsequent characterization.

### 3.4. Characterization

Electromagnetic wave (EMW) absorption properties were evaluated using a vector network analyzer (Ceyear 3674G, Ceyear Technologies Co., Ltd., Qingdao, China) via the coaxial line method. The product powders (30 wt%) were uniformly blended with paraffin wax (70 wt%) and pressed into toroidal-shaped rings with an outer diameter of 7.0 mm, an inner diameter of 3.0 mm, and an average thickness of approximately 2.0 mm under 2 MPa pressure. The complex permittivity, complex permeability, reflection loss, and impedance matching were simultaneously determined over the frequency range of 2–18 GHz with 400 data points. X-ray diffraction (XRD) patterns were acquired using a Bruker D8 Advance diffractometer (Bruker Corporation, Billerica, MA, USA) with a scanning rate of 4 °/min, a step size of 0.02°, and a 2θ range of 5–90°. All XRD results were normalized by total area intensity. Scanning electron microscopy (SEM) and energy-dispersive X-ray spectroscopy (EDS) were performed on a ThermoFisher Apreo 2S microscope (Thermo Fisher Scientific, Waltham, MA, USA). Thermogravimetric analysis (TGA) was conducted on a Mettler Toledo TGA/DSC 3+ instrument (Mettler-Toledo GmbH, Switzerland). Samples were placed in 70 μL alumina crucibles and heated from 30 to 1000 °C at 5 °C/min under argon flow, followed by a 5 min isothermal hold at 1000 °C.

## 4. Conclusion

In conclusion, this study successfully demonstrates a cation engineering strategy for tailoring the electromagnetic wave absorption of ZIF-67-derived composites through controlled element substitution (Fe, Mn, Zn, Ni) and stoichiometric variation. We have shown that the exceptional performance of the CoNi14 composite stems from synergistic magnetic coupling and interface polarization, while Mn/Zn doping enhances dipole polarization through lattice distortion. Critically, we identified that a higher Co/X ratio is key to preserving favorable core–shell morphologies for optimal impedance matching, whereas Fe^2+^ incorporation leads to excessive conductivity and impedance mismatch. The two principal advancements of this work are the establishment of element-specific doping thresholds for balanced loss mechanisms and the decoupling of permittivity/permeability contributions. As a result, superior broadband performance (EAB = 4.52 GHz at 1.8 mm, RL_min_ = −24.5 dB) is achieved in the CoNi14 sample. This work provides a valuable materials genome database, paving the way for designing next-generation, high-performance absorbers for advanced 5G/6G shielding and radar stealth technologies.

## Figures and Tables

**Table 1:** Molar ratio of substance in synthesis process.

Substance	Molar Ratio (ZIF-67:X)	Abbreviation
FeSO_4_·7H_2_O	1:4 (2.752 mmol)	CoFe14
FeSO_4_·7H_2_O	1:1 (0.688 mmol)	CoFe11
Mn(NO_3_)_2_·4H_2_O	1:4 (2.752 mmol)	CoMn14
Mn(NO_3_)_2_·4H_2_O	1:1 (0.688 mmol)	CoMn11
Zn(NO_3_)_2_·6H_2_O	1:4 (2.752 mmol)	CoZn14
Zn(NO_3_)_2_·6H_2_O	1:1 (0.688 mmol)	CoZn11
Ni(NO_3_)_2_·6H_2_O	1:4 (2.752 mmol)	CoNi14
Ni(NO_3_)_2_·6H_2_O	1:1 (0.688 mmol)	CoNi11

**Table 2:** EABs of LDH materials.

Materials	EAB_max_/GHz	Maximum/dB	Thickness at EAB_max_/mm	EAB Frequency Range/GHz
Co:Fe = 1:4	—	−8.85	—	—
Co:Fe = 1:1	0.24	−18.30	8.0	15.88–16.12
Co:Mn = 1:4	4.29	−15.84	2.0	10.70–14.99
Co:Mn = 1:1	4.33	−16.70	1.5	12.35–16.68
Co:Zn = 1:4	2.57	−16.76	5.0	15.43–18.00
Co:Zn = 1:1	3.53	−20.44	2.0	11.14–14.67
Co:Ni = 1:4	4.52	−24.50	2.0	13.48–18.00
Co:Ni = 1:1	—	−7.60	—	—

## Data Availability

The original contributions presented in this study are included in the article. Further inquiries can be directed to the corresponding authors.
